# Estimation of Genetic Parameters for Female Fertility Traits in the Polish Holstein-Friesian Population

**DOI:** 10.3390/ani12121485

**Published:** 2022-06-08

**Authors:** Agnieszka Otwinowska-Mindur, Ewa Ptak, Wojciech Jagusiak, Andrzej Zarnecki

**Affiliations:** 1 Department of Genetics, Animal Breeding and Ethology, University of Agriculture in Krakow, al. Mickiewicza 24/28, 30-059 Krakow, Poland; ewa.ptak@urk.edu.pl (E.P.); wojciech.jagusiak@urk.edu.pl (W.J.); 2 National Research Institute of Animal Production, ul. Krakowska 1, 32-083 Balice, Poland; rzzarnec@cyf-kr.edu.pl

**Keywords:** fertility, genetic parameters, Holstein-Friesian

## Abstract

**Simple Summary:**

Female fertility is an important functional trait in dairy cattle. The aim of this study was to estimate genetic parameters for some fertility traits of Polish Holstein-Friesian cows. The results indicate that a few analyzed traits could be of use in genetic evaluation of Polish Holstein-Friesian cows.

**Abstract:**

The objective of this study was to estimate genetic parameters for the analyzed fertility traits of Polish Holstein-Friesian primiparous and multiparous cows, as a step toward the incorporation of new traits into routine genetic evaluation. Lactation records of 116,836 Polish Holstein-Friesian cows were studied. The records cover the first three lactations of all cows. The cows, daughters of 2884 sires, were calved from 2006 to 2020. The conception rate for heifers (CRh) and for cows in the second (CR2) and third parity (CR3), the interval from first calving to first insemination (CTFI), the days open (DO), and the interval from first to successful insemination for heifers (FSh) and for cows in second (FS2) and third (FS3) parity were analyzed. The BLUPf90 package and a Bayesian method via Gibbs sampling were used to estimate (co)variance components. In general, all heritabilities were low and ranged from 0.013 (CTFI) to 0.038 (FS2). The heritability of conception rate and interval from first to successful insemination was slightly lower for heifers than for cows. Genetic correlations were moderate to high with two exceptions: correlation of CTFI with CRh (0.033) and with FSh (−0.051). The results indicate that a few analyzed traits could be used in genetic evaluation of Polish Holstein-Friesian cows. It is suggested to complement the selection index with traits for both heifers and cows, such as the interval from first to successful insemination (i.e., FSh, FS2 and FS3), in order to enable effective improvement of female fertility scores in the Polish Holstein-Friesian population.

## 1. Introduction

Female fertility is an important functional trait in dairy cattle. Poor fertility affects milk production and reproduction costs through higher culling rate, costs of fertility treatments, higher number of inseminations per cow, and longer calving intervals. All the mentioned causes decrease herd profitability [1,2,3]. Environmental factors, such as herd management, year and month, age at calving, and age at insemination have been found to affect fertility traits [4,5].

The traditional breeding goal in dairy cattle was to obtain high-yielding cows with constantly increased milk, fat, and protein yields. Long-lasting selection for increased milk production has led to a decline in fertility in dairy cows as a result of an unfavourable genetic relationship between production and fertility [1,4,6,7,8,9]. Fertility, a trait of economic importance to dairy farmers, has been suggested by many breeding organizations for inclusion into the selection index for years. The main problem in doing that has been to find the relevant, precise measures of female fertility traits, independent of farm management decisions. Another problem is poor recording practice on dairy farms: some breeders recorded all insemination events, whereas others recorded only the last insemination date, omitting unsuccessful services. In recent years, none of the modern selection indices are based only on milk production traits. They also include functional traits, such as type traits, female fertility traits, and longevity, and they have been revised periodically by including new traits valuable to farmers, i.e., the traits favouring development of efficient, healthy, and fertile cows [10]. Updates of selection indices have reflected not only changing economic conditions but also changing genetic parameters among traits. Milk production traits make up about 50% of the total indexes [9]. For example, in Poland the milk production traits comprise 40% of the total index, whereas the fertility component has 15% weight in the total merit index [11]. This change in dairy selection has been beneficial, as it reverses the decline in reproductive traits, as well as some functional traits in dairy cattle [7,9]. Many countries have included novel fertility traits into selection schemes for decades [12,13] but many have not, and there has been growing interest in genetic selection to improve cow health and welfare [14]. Many studies indicate that heifer fertility traits are more heritable than those for cows, and that fertility can change with the age of the cow [4]. To better evaluate the reproductive ability of cows, fertility traits of both heifers and cows should be considered simultaneously in the total selection index. The most common traits used in breeding programs are calving interval, days open, calving to first service interval, interval from first to successful insemination, non-return rate to 56 days, number of inseminations per conception, conception rate, age at first calving, and age at first service [3,7,8,15]. Some of these traits have been part of the Interbull routine genetic evaluation for females’ fertility traits. The Polish national genetic evaluation system includes the following four fertility female traits: conception rates for heifers and for cows, interval from first calving to first insemination, and days open [15]. Recently the inclusion of interval from first to successful insemination for both heifers and cows has been considered in Poland, because of international harmonization of fertility traits. When evaluating new traits in order to include them in a breeding program, it is worth to check how strongly they are correlated with traits already included in the selection. Relatively low relationships of used with new traits indicate that the latter may provide information not available from existing traits. Otherwise, observations for new traits probably provide little additional information important for selection [10]. The objective of this study was to estimate genetic parameters for some fertility traits of Polish Holstein-Friesian primiparous and multiparous cows, as a step toward the incorporation of themthe interval from first to successful insemination into the national genetic evaluation system.

## 2. Materials and Methods

Lactation records from the first three lactations of 116,836 Polish Holstein-Friesian cows were analysed. The data came from the Polish national recording system (SYMLEK) and were made available by the Polish Federation of Cattle Breeders and Dairy Farmers. The cows, daughters of 2884 sires, and 107,573 dams among which 17,370 have own fertility data, so the pedigree file consistent 209,923 animals. The cows were calved from 2006 to 2020 at age 18–48 months in the first, 29–65 months in second, and 41–75 months in third parity. Two seasons of calving were assumed (October–March and April–September).

The following fertility traits were analysed:

(1) CR [%]—conception rate for heifers (CRh) and for cows in second (CR2) and third (CR3) parity. Conception rates were coded as follows: CR = 100 × 1/number of all inseminations, i.e., the CR is a probability of conception expressed in percent. The CR = 100 when the first insemination was successful. If more than 15 inseminations were made or if no insemination was successful, CR = 100/16 = 6.25 [11];
(2) CTFI [days]—interval from first calving to first insemination;
(3) DO [days]—days open, i.e., interval between first calving and conception;
(4) FS [days]—interval from first to successful insemination for heifers (FSh) and for cows in the second (FS2) and third (FS3) parity.

The CR, CTFI and DO were defined according to Polish national genetic evaluation system and FS was a new trait planned to be included into national genetic evaluation system. Only DO and CTFI were normally distributed traits, whereas CR and FS were not.

Data were restricted to a minimum 10 daughters per sire and a minimum of 3 contemporaries per herd-year of first calving or first insemination subclasses. More than 90% of half-sib family groups in the restricted file comprised 10–99 cows (Table 1).

The BLUPf90 package and a Bayesian method via Gibbs sampling were used to estimate (co)variance components [16]. The linear model used for CTFI and DO was as follows:

$$\begin{array}{c} Y_{ijklm}=a_{i}+HY_{j}+M_{k}+\beta \;\cdot \;AFC_{l}+\epsilon_{ijklm} \end{array}$$

where $Y_{ijklm}$—CTFI or DO for *i*-th cow calved for the first time in *j*-th herd-year (HY) subclass in *k*-th month of year of first calving (*Y*) at *l*-th age (AFC); $a_{i}$—random additive genetic effect (with 209,923 levels); $HY_{j}$—fixed herd-year of first calving effect (with 13,994 levels); $M_{k}$—fixed month of year of first calving effect (with 12 levels); β—linear regression of *Y* on age at first calving ($AFC_{l}$); $AFC_{l}$—age at first calving (18–48 months); $\epsilon_{ijklm}$—residual effect.

The following linear model for CR and FS was used:

$$\begin{array}{c} Y_{ijklm}=a_{i}+HYFI_{j}+M_{k}+\beta \;\cdot \;AFI_{l}+\epsilon_{ijklm} \end{array}$$

where $Y_{ijklm}$—CRh, CR2, CR3, FSh, FS2, or FS3 for *i*-th cow insemination for the first time in *j*-th herd-year (HYFI) subclass in *k*-th month of year of first insemination (*Y*) at *l*-th age (AFI); $a_{i}$—random additive genetic effect (with 209,923 levels); $HYFI_{j}$—fixed herd-year of first insemination effect (with 13,992, 13,997, and 14,033 levels in the first, second, and third parity, respectively); $M_{k}$—fixed month of year of first insemination effect (with 12 levels); β—linear regression of *Y* on age at first insemination ($AFI_{l}$); $AFI_{l}$—age at first insemination (9–40, 20–60, and 30–70 months in first, second and third parity, respectively); $\epsilon_{ijklm}$—residual effect.

The number of generated samples of (co)variance components was equal to 100,000, with the first 5000 samples discarded as the burn-in based on the plot of Gibbs samples. Only every 100th sample was written for use in further calculations.

## 3. Results

Descriptive statistics of all female fertility traits are shown in Table 2. Mean CR was highest for heifers (84%) and decreased by more than 10% in second (73%) or third (70%) parity. The mean length of CTFI was about 89 days, and of DO about 121 days, with a broad range of values, from 20 to 700 for both traits. Mean FS for heifers (FSh) was about 16 days and was less than half of FS for cows in second (FS2 = 33 days) or third (FS3 = 37 days) parity. Standard deviations for CR and FS increased with parity.

The heritability of all analysed fertility female traits is presented in Table 3. In general, all heritabilities were low and ranged from 0.013 (CTFI) to 0.038 (FS2). Standard deviations for heritability were between 0.004 and 0.025. Heritability of CR was slightly lower for heifers (0.021) than for cows (0.029). A similar trend was observed for heritability of FS; the heritability of FSh was 0.019 and for cows was greater than 0.03, with a higher difference between first and later parities when compared with the values of CR heritability.

Table 4 shows genetic and phenotypic correlations among eight fertility female traits. Genetic correlations were moderate to high with two exceptions: the correlation of CTFI with CRh (0.033) and with FSh (−0.051). A high positive genetic correlation was estimated between DO and FS2 (0.972). In turn, high negative correlations (>0.92, ignoring sign) were found between DO and CR2 and between CR and FS within the first, second, and third parity. The correlation between DO and CR2 indicates that the longer the time from first calving to conception, the longer the interval between first and successful insemination before second calving. The correlation of CR with FS means that the longer interval between first and successful insemination, the lower the CR both for heifers and for cows. CTFI was weakly genetically correlated with two traits of heifers: CRh (0.033) and FSh (−0.051). The genetic correlation was also low between CTFI and CR2 (−0.327) and CTFI and CR3 (−0.276), showing that a long period from first calving to first insemination afterwards was connected with low CR in second and third parity.

Most of the phenotypic correlations were low to moderate and in most cases they were smaller than the genetic ones (Table 4). High phenotypic correlations were estimated for the same pairs of traits that were highly genetically correlated: a high positive phenotypic correlation between DO and FS2 (0.947), and negative (>0.8, ignoring sign) between DO and CR2, CRh and FSh, CR2 and FS2, and CR3 and FS3. Phenotypic correlations close to zero were estimated for CR3 with CR2, CTFI, DO, FSh or FS2, and FS3, as well as for CRh, CR2, CTFI, and FSh.

## 4. Discussion

Mean CR was highest in the first parity (84%) and decreased in subsequent parities by about 14% to 70.44% in the third parity. Yamazaki et al. [17], who defined CR as a binary trait, found CR for first lactation between 36.5% and 39.3%, depending on the housing system; and about 35% in the second and third parity, with minor differences among housing systems. Tsuruta et al. [18] and Aguilar et al. [19] also presented mean CR lower than our results. Additionally, Tsuruta et al. [18] observed that CR in small herds (34.4%) was 4.9% higher than in large herds (29.5%). They concluded that artificial insemination was conducted in earlier stages of lactation in large herds than in small herds.

CTFI indicates the recovery of an animal’s ability to recycle after calving. Additionally, days to first service is an indicator of post-partum return to reproductive function [6,8]. In the present study, mean CTFI was about 89 days, approximately 10 days longer than CTFI reported earlier by Jagusiak and Zarnecki [20] and by Rzewuska and Strabel [21] for Polish Holstein-Friesian cows. Similar results for CTFI were presented for the Canadian population [4,22], whereas slightly lower values for CTFI were reported for United Kingdom Holsteins [1], Spanish Holsteins [2,6], and Chinese Holsteins [8].

Days open, that is, the interval from calving to conception, is the sum of CTFI and FSh; it is an important, useful trait because it can be calculated easily from milk recording data, whereas other fertility traits require data on inseminations and pregnancy, which are not available in many recording systems [8]. Additionally, the DO and CTFI are a traits influenced by farmer’s decisions about the length of voluntary waiting period, the efficiency of estrus detection and the application of synchronization products. The days open is the only trait independent of the effectiveness of insemination and pregnancy diagnosis [21]. Mean DO in the Polish Holstein-Friesian population was about 121 days, what is consisntent with results of Rzewuska and Strabel [21], but 11 days shorter than previously reported average [20]. Mean DO in some studies was similar to our result [3,8,21,22]; DO values in other reports were lower, indicating a shorter (i.e., more preferable) period between calving and conception [2,6,18,23].

Another fertility trait, the interval from first to successful insemination (FS), has a great range of values both for heifers (FSh = 0 to 165 days) and for cows (FS2, FS3 = 0 to 237 days). On average, the number of days between first and last insemination for heifers was about 16 days, and for cows in second and third lactation increased to 33 and 37 days, respectively. Similar results for FS were obtained for the Canadian Holstein population [4,22]. Longer intervals between first service and conception in heifers and cows were reported by Kadarmideen et al. [1], Brzáková et al. [3], Toledo-Alvarado et al. [23] and Muuttoranta et al. [24]. Kadarmideen et al. [1] obtained a decreasing tendency for FS in subsequent lactations of the United Kingdom Holstein population. The opposite trend was observed for Polish Holstein-Friesian cows in the present study what is agreement with results of Muuttoranta et al. [24] for Nordic Holstein-Friesian cows.

Estimated heritability for female fertility traits was generally low (0.013–0.038) and consistent with the results from other studies [1,3,4,6,7,8,20,21,24,25]. The low heritability of female fertility traits indicated the high influence of herd, management, and other environmental effects on these traits [1,3,5,24]. However, low heritability did not necessarily mean that there was not enough genetic variability to justify selection for those traits. The heritability of CR varied from 0.021 for heifers to 0.029 for cows, slightly higher than in studies by Yamazaki et al. [17] and Aguilar et al. [19]. Compared to our results Muuttoranta et al. [24] estimated lower heritabilities of CR for heifers (0.008) and similar for cows (0.025–0.030). In turn, heritability for CR estimated by Tsuruta et al. [18] was higher than ours: 0.052 in large herds and 0.057 in small herds. Heritability of CTFI (0.013) and DO (0.037) was lower than in some previous studies [6,8,22,26]. Heritability of DO obtained in this study was similar to results from Brzáková et al. [3] and slightly higher than obtained by Liu et al. [7]. Rzewuska and Strabel [21] estimated higher heritability of CTFI (0.055) and DO (0.049) in Polish Holstein-Friesian primiparous cows. Jagusiak and Zarnecki [20] also presented slightly higher heritability of DO in Polish Holstein-Friesian population for the first, second, and third parity: 0.051, 0.045, and 0.043, respectively.

Heritability of FS increased slightly with subsequent parity, from 0.019 for heifers to 0.031–0.038 for cows. Brzáková et al. [3] observed a similar though lower trend in the Czech Holstein population, where heritability of FS increased from 0.010 to 0.025; they wrote that the interval from first service to conception (FS) was less influenced by farmers’ decisions than traits, such as days open (DO) or calving interval (CI), because if a farmer decided to inseminate a cow he continued it until the cow became pregnant. Heritability of FS in Canadian Holsteins also increased with parity number, from 0.01 to 0.03 [22] or from 0.03 to 0.07 [4]. Muuttoranta et al. [24] estimated heritability of FS for Nordic Holsteins and they also observed a similar, upward trend. The FS for heifers was 0.012 and for cows varied from 0.041 to 0.048. Liu et al. [7] presented the opposite trend in heritability of FS estimated jointly for three breeds in three countries (Germany, Austria and Luxembourg): slightly higher heritability (0.014) for heifers and lower heritability (0.010) for cows. Much higher heritability of FS was calculated by Jagusiak and Zarnecki [20] for Polish Holstein-Friesian heifers (0.092) and for cows in the second (0.086) and third (0.054) parity. In turn, Rzewuska and Strabel [21] estimated heritability of FS (0.034) for Polish Holstein-Friesian primiparous cows higher than our but lower than Jagusiak and Zarnecki [20]. Kadarmideen et al. [1] wrote that a loss of pregnancy success at the first insemination could be the result of parturition and post-parturition incidents. The weight on female fertility traits in the selection index for the Polish Holstein-Friesian population is 15% [11]. The low emphasis and low heritability of fertility traits have caused dairy producers not to expect rapid genetic improvement for these traits in the short term [5].

Among genetic correlations estimated in this study a high and positive genetic relationship was observed between DO and FS2 (0.972), indicating that animals would rank similarly for DO and FS2. This means that genetic improvement of one of these traits, for example DO, could cause a correlated response in the second correlated trait. In turn, high but negative genetic correlations were estimated between DO and CR2, as well as between CR and FS within each parity (CRh and FSh, CR2 and FS2, and CR3 and FS3). A favourable genetic correlation between CR and FS means that genetic selection for improvement of CR would result in increased FS both for heifers and for cows. Additionally, a negative correlation between CR and FS within parity is biologically favourable, because a short FS period is linked to desirable CR value [24]. Kadarmideen et al. [1] noted that a high genetic correlation among some fertility traits was expected because various fertility measurements were linked and could be calculated as a function of other traits. Low or moderate and positive correlations between CR for heifers and CR for cows in second and third parity were estimated in this study. Higher and positive genetic correlations (0.732–0.877) among CR in the first three parities were estimated by Aguilar et al. [19]. The genetic correlation between CTFI and DO presented in this paper was in close agreement with the result (0.58) of Guo et al. [8] but lower than the result (0.88) for Canadian Holsteins [22]. CTFI and FSh were not genetically correlated (−0.051) whereas the genetic correlation between CTFI and FS for cows was moderate (0.43). Jamrozik et al. [4] estimated stronger genetic relationship (0.66) between the latter two traits. Low to moderate genetic correlations between CTFI and FS for heifers (0.15) or cows (0.60) were found in Canadian Holsteins [22]. The moderate genetic correlations between DO and FSh (0.48) and between DO and FS3 (0.65), estimated in this study were higher than estimated by Jagusiak and Zarnecki [20] between FSh and DO (0.32). A slightly lower genetic relationship between DO and FSh (0.35) and a slightly higher one between DO and FS for cows (0.88) was presented for Canadian Holsteins [22]. According to the results for the Czech Holstein population given by Brzáková et al. [3], DO and FSh were practically genetically independent (0.008), whereas the genetic correlation of DO with FS for cows (FS2, FS3) was close to one, indicating that these traits were genetically almost the same. The genetic correlation values indicated that the majority of fertility traits for heifers and cows were not the same genetically. For instance, the genetic relationship between CRh and CR for cows (CR2, CR3), as well as for FSh and FS for cows was moderate and not higher than 0.52. These two traits (CR and FS) were strongly genetically correlated only in the second and third parity (0.64–0.70). Oliveira Junior et al. [5] emphasized that the currently measured fertility traits are highly influenced by management decisions and human error: for example, voluntary waiting periods, data recording errors, failure to record events, and to detect oestrus. Mismanagement (e.g., inseminating animals at an inappropriate point in the oestrus cycle) or unrecorded management decisions (e.g., unrecorded hormonal synchronization treatments) also strongly affected female fertility traits [5].

## 5. Conclusions

In summary, the heritability estimates of eight fertility traits in the Polish Holstein-Friesian population were low. Strong positive genetic and phenotypic correlations were observed between DO and FS2, while strong negative correlations, both genetic and phenotypic, were estimated between DO and CR2, CRh and FSh, CR2 and FS2, and CR3 and FS3. Genetic evaluation and selection of animals with high genetic merit for fertility traits could improve the level of heifer and cow fertility.

The results indicate that some analysed traits could be of use in genetic evaluation of Polish Holstein-Friesian cows. It is suggested to complement the selection index by incorporating traits for both heifers and cows, such as interval from first to successful insemination, to enable effective improvement of female fertility scores in the Polish Holstein-Friesian population. All fertility traits typically are lowly heritable so the expected breeding progress may be slow and not sufficient. Therefore, to enhance genetic gain in cows’ reproduction, the functional traits more highly heritable and relatively strongly correlated with fertility traits should be included into selection index. Additionally, in the future genomic evaluation of female fertility traits, which helps to predict breeding values more precisely, is considered to improve genetic progress in fertility of cows.

## Figures and Tables

**Table 1:** Distribution of size of progeny groups in the file restricted to 10 daughters per sire.

Number of Daughters	Number of Sires
20–49	862
50–99	296
100–199	148
200–499	74
≥500	15
Total	2884

**Table 2:** Descriptive statistics of female fertility traits.

Trait ^1^	Mean	SD ^2^	Min	Max	CV [%] ^3^
CRh [%]	84.09	25.76	8.33	100	30.6
CR2 [%]	73.11	30.81	7.69	100	42.1
CR3 [%]	70.44	31.46	8.33	100	44.7
CTFI [day]	88.92	43.91	20.00	700	49.4
DO [day]	121.20	64.36	20.00	700	53.1
FSh [day]	15.98	30.30	0.00	165	189.6
FS2 [day]	33.43	49.66	0.00	237	148.5
FS3 [day]	37.36	50.97	0.00	220	136.4

^1^ CRh—conception rate for heifers, CR2—conception rate for cows in second parity, CR3—conception rate for cows in third parity, CTFI—interval from first calving to first insemination, DO—days open, FSh—interval from first to successful insemination for heifers, FS2—interval from first to successful insemination for cows in second parity, and FS3—interval from first to successful insemination for cows in third parity; ^2^ SD—standard deviation; ^3^ CV [%]—coefficient of variation.

**Table 3:** Estimated genetic ($\sigma_{G}^{2}$) and residual ($\sigma_{R}^{2}$) variance, and heritability ($h^{2}$) of fertility female traits.

$\sigma_{G}^{2}$			$\sigma_{R}^{2}$		$h^{2}$	
**Trait ^1^**	**Mean**	**SD** ^2^	**Mean**	**SD ^2^**	**Mean**	**SD ^2^**
CRh	16.66	4.27	757.37	85.32	0.021	0.004
CR2	117.25	55.69	5375.46	4233.72	0.029	0.018
CR3	27.63	5.63	933.49	12.01	0.029	0.006
CTFI	21.68	23.86	1712.99	174.70	0.013	0.015
DO	176.49	92.82	5097.94	1074.06	0.037	0.025
FSh	17.55	4.44	898.75	28.39	0.019	0.005
FS2	135.99	60.73	3827.22	1161.70	0.038	0.025
FS3	73.92	12.96	2329.90	14.23	0.031	0.005

^1^ CRh—conception rate for heifers, CR2—conception rate for cows in second parity, CR3—conception rate for cows in third parity, CTFI—interval from first calving to first insemination, DO—days open, FSh—interval from first to successful insemination for heifers, FS2—interval from first to successful insemination for cows in second parity, and FS3—interval from first to successful insemination for cows in third parity; ^2^ SD—standard deviation.

**Table 4:** Genetic (above diagonal) and phenotypic (below diagonal) correlations of fertility female traits.

CRh			CR1		CR2		CTFI		DO		FSh		FS1		FS2	
**Trait ^1^**	**Mean**	**SD ^2^**	**Mean**	**SD ^2^**	**Mean**	**SD ^2^**	**Mean**	**SD ^2^**	**Mean**	**SD ^2^**	**Mean**	**SD ^2^**	**Mean**	**SD ^2^**	**Mean**	**SD ^2^**
CRh			0.499	0.150	0.287	0.110	0.033	0.134	−0.426	0.177	−0.961	0.030	−0.462	0.161	−0.207	0.121
CR2	0.258	0.113			0.696	0.082	−0.327	0.172	−0.923	0.086	−0.554	0.150	−0.971	0.022	−0.689	0.130
CR3	0.035	0.008	0.092	0.014			−0.276	0.110	−0.601	0.092	−0.318	0.106	−0.606	0.092	−0.936	0.022
CTFI	−0.120	0.102	−0.369	0.190	−0.042	0.012			0.572	0.195	−0.051	0.160	0.427	0.160	0.426	0.104
DO	−0.261	0.126	−0.891	0.096	−0.088	0.013	0.646	0.041			0.479	0.171	0.972	0.043	0.650	0.130
FSh	−0.872	0.015	−0.290	0.127	−0.034	0.008	0.142	0.114	0.298	0.140			0.524	0.149	0.247	0.123
FS2	−0.275	0.117	−0.943	0.047	−0.090	0.013	0.403	0.197	0.947	0.058	0.312	0.132			0.636	0.131
FS3	−0.032	0.009	−0.099	0.018	−0.841	0.003	0.060	0.013	0.103	0.014	0.034	0.009	0.101	0.016		

^1^ CRh—conception rate for heifers, CR2—conception rate for cows in second parity, CR3—conception rate for cows in third parity, CTFI—interval from first calving to first insemination, DO—days open, FSh—interval from first to successful insemination for heifers, FS2—interval from first to successful insemination for cows in second parity, and FS3—interval from first to successful insemination for cows in third parity; ^2^ SD—standard deviation.

## Data Availability

Restrictions apply to the availability of these data. Data were obtained from the Polish Federation of Cattle Breeders and Dairy Farmers.
